# Society Meetings

**Published:** 1907-05

**Authors:** 


### SOCIETY MEETINGS.

The eighth meeting of the Society of Clinical Surgery was held at Buffalo and Cleveland on April 19 and 20, 1907. Of the 40 members of the society thirty were present at Buffalo. The program for the meeting was as follows:

BUFFALO, FRIDAY, APRIL 19

Local Committee, Dr. Roswell Park

The Iroquois Hotel, headquarters for the meeting.

9.30 A.M.—Buffalo General Hospital, Surgical Amphitheatre.—Dr. Roswell Park and Colleagues (by invitation): Demonstrations of Cases, and Operations. 1 P.M.—Luncheon at the University Club (by invitation of Dr. Park). 2 P.M.—State Cancer Laboratory.—Drs. Gaylord and Cowles (by invitation): Demonstration of recent work in the investigation of Cancer. 3 P.M.—University Medical School.—Dr. H. W. Williams (by invitation) and Dr. F. C. Busch (by invitation): Demonstration of recent experimental work on the artificial passage of Gall-stones with Infection of the Duodenum. 3.30 P.M.—Dr. F. C. Busch (by invitation): Transplantation of the Adrenals. 4 P.M.—Dr. J. A. Gibson (by invitation): Exhibition of specimens showing abnormalities and anomalies in the Sphenoidal Sinus. Dr. Park exhibited a series of pictures illustrating medicine and surgery in classic art, and in caricature. 7 P.M.—Dinner and business meeting at the Buffalo Club.

CLEVELAND, SATURDAY, APRIL 20

Local Committee, Dr. G. W. Crile

9 A.M.—Lakeside Hospital, Surgical Amphitheatre.—Dr. George W. Crile, Dr. Dudley P. Allen (by invitation), Dr. F. E. Bunts (by invitation), Dr. C. E. Briggs (by invitation), Dr. Hunter Robb (by invitation), and Dr. Henry A. Becker (by invitation): Clinical problems, illustrated by cases and operations.—Luncheon at Lakeside Hospital. 1.30 P.M.—Western Reserve Medical College.—Dr. C. A. Hamann (by invitation): Demonstrations in Surgical Anatomy of the Veins of the Neck, and Pathological Specimens. Dr. Torald Sollman (by invitation): Pharmacological Demonstrations. Dr. J. J. R. MacLeod (by invitation): Physiological Demonstrations. Dr. W. E. Lower (by invitation): On the Control of Hemorrhage in Operations on the Kidney. Dr. G. W. Crile and Dr. Hitchings (by invitation): The Technique of Direct Transfusion of Blood. Dr. G. W. Crile and Drs. Haskins, Folin, Cole (by invitation): Determinations of the Metabolism in Transfused (a) Dogs, (b) Human subjects. Dr. G. W. Crile and Dr. Dooley (by invitation): The Phenomena and Treatment of Acute Hemorrhage. Dr. G. W. Crile and Drs. Lenhart, Hitchings, and Eisenbrey (by invitation): Can a Differential Diagnosis between a Progressive Hemmorrhage and Shock be made by Blood Examinations? Dr. G. W. Crile and Dr. Dolley (by invitation): (a) Strychnine Poisoning; Diphtheria Toxemia. Dr. G. W. Crile and Dr. Lenhart (by invitation): (b) Illuminating-gas Poisoning. Dr. G. W. Crile and Dr. Cole (by invitation): (c) In Uremia. Dr. G. W. Crile and Drs. Dolley, Lenhart, and Cole (by invitation): Transfusion in Surgical Shock. Dr. G. W. Crile and Drs. Dolley, Lenhart, and Cole (by invitation): Further Studies in Resuscitation and Brain Anemia.

---

The Association of American Medical Colleges will hold its annual meeting at the Hotel Raleigh, Washington, D. C., Monday,

May 6, 1907, beginning at 10 a. m. Dr. George M. Kober, of Washington, is the president. Dr. Eli H. Long, of Buffalo, will open the discussion on Teaching Materia Medica, Pharmacology and Therapeutics, a paper to be presented by Dr. Torald Sollman, of Cleveland.

---

The Buffalo Academy of Medicine held meetings during the month of April as follows:

*Section on Surgery.*—Tuesday evening, April 2, program: (a) Typhoid perforation, John Parmenter; (b) Intestinal obstruction, Marshall Clinton.

*Section on Medicine.*—Tuesday evening, April 9, Program: A consideration of some of the physical signs in the chest of infants and children, S. McC. Hamill, Philadelphia, discussion was opened by Irving M. Snow.

*Postponement of Meeting.*—The meeting of the section of Pathology announced in the program for Tuesday evening, April 16, was postponed until Tuesday, April 30.

*Section on Obstetrics and Gynecology.*—Tuesday evening, April 23, Program: (a) Delayed lactation, George A. Himmelsbach. (b) Conservative operations on the tubes, Edward C. Mann.

---

The American Medical Editors' Association will hold its annual meeting at Atlantic City on Saturday, June 1, and Monday June 3, 1907, with headquarters at the Marlborough-Blenheim Hotel. James Evelyn Pilcher, is the president, who will deliver an address on "The future of Medical Journalism." Joseph MacDonald, Jr., New York, is the secretary.

---

The Western New York Homeopathic Medical Society, at its twenty-third annual session held at Rochester, April 12 and 13, 1907, elected the following named officers: president, W. H. Doane, Rochester; first vice-president, Frank B. Seitz, Buffalo; second vice-president, Joseph Rieger, Dunkirk; secretary-treasurer, Clayton W. Seamon, Buffalo; censors, W. Louis Wilson, Niagara Falls, Warren C. Day, Rochester, William A. Keegan, Rochester, George S. Price, Fairport, John W. Baker, Batavia.

---

The regular meeting of The Medical Society of the County of Erie was held Monday, April 8, 1907, at 8.45 P.M., under the presidency of Dr. A. H. Briggs, of Buffalo. The following papers were presented: The need of a trained microscopist in every community, William C. Krauss; Gun-shot wounds of the body, Vertner Kennerson; The physician in the wild and wooley west, George E. Fell.

The National Confederation of State Medical Examining and Licensing Boards will hold its seventeenth annual meeting at Atlantic City on Tuesday, June 4, 1907. The place at which the meeting will be held will be announced in due season. A varied and interesting program is being prepared. Dr. Murray Galt Motter, of Washington, D. C., is the secretary.

The Thirty-second Meeting of the American Academy of Medicine (Specialising in Medical Sociology) will be held at the Hotel Dennis, Atlantic City, on Saturday, June 1, and Monday, June 3, 1907, under the presidency of Dr. Casey A. Wood, of Chicago.

The American Climatological Association will hold its twenty-fourth annual meeting at the Willard, Washington, D. C., May 7-9, 1907, under the presidency of Dr. Thomas Darlington, president of the board of health of New York city.

